# The Immune System Is a Natural Target for Estrogen Action: Opposing Effects of Estrogen in Two Prototypical Autoimmune Diseases

**DOI:** 10.3389/fimmu.2015.00635

**Published:** 2016-01-06

**Authors:** Deena Khan, S. Ansar Ahmed

**Affiliations:** ^1^Department of Biomedical Sciences and Pathobiology, Virginia-Maryland College of Veterinary Medicine, Virginia Tech, Blacksburg, VA, USA

**Keywords:** estrogen, autoimmune, SLE, MS, signaling, immune cell

## Abstract

Analogous to other physiological systems, the immune system also demonstrates remarkable sex differences. Although the reasons for sex differences in immune responses are not precisely understood, it potentially involves differences in sex hormones (estrogens, androgens, and differential sex hormone receptor-mediated events), X-chromosomes, microbiome, epigenetics among others. Overall, females tend to have more responsive and robust immune system compared to their male counterparts. It is therefore not surprising that females respond more aggressively to self-antigens and are more susceptible to autoimmune diseases. Female hormone (estrogen or 17β-estradiol) can potentially act on all cellular subsets of the immune system through estrogen receptor-dependent and -independent mechanisms. This minireview highlights differential expression of estrogen receptors on immune cells, major estrogen-mediated signaling pathways, and their effect on immune cells. Since estrogen has varied effects in female-predominant autoimmune diseases such as multiple sclerosis and systemic lupus erythematosus, we will mechanistically postulate the potential differential role of estrogen in these chronic debilitating diseases.

## Introduction

Although the principal function of sex steroid action is to regulate reproductive functions, studies in diverse fields have unequivocally established that sex steroids also act on diverse non-reproductive tissues include immune, central nervous, cardiovascular, and skeletal systems, as well as cells from liver, skin, and kidneys (1–5). In this brief review, we will discuss estrogen effects on the immune cells, the estrogen-specific receptor expression on cells of the immune system, and key estrogen-receptor mediated-signaling pathways involved. In addition, we will analyze the differential response of estrogen in two typical female-predominant autoimmune diseases.

## Estrogen Regulation of Cells of the Innate and Adaptive Immune System

There is now an enormous amount of literature on estrogen’s effects on the cells of the innate immune system [neutrophils, macrophages/monocytes, natural killer cells, dendritic cells (DC)], and the adaptive immune system (T and B cells). These aspects have been comprehensively covered in many reviews (5–7) and hence are beyond the scope of this minireview. Estrogens have been shown to regulate neutrophil numbers and functions that include chemotaxis, infiltration, production of superoxide anion and myeloperoxidase, induction of chemokines (cytokine induced neutrophil chemoattractants such as CINC-1, CINC-2β, and CINC-3, monocyte chemoattractant protein-1), and cytokines (e.g., TNF-α, IL-6, IL-1β) (8–10). Table 1 shows key selected genes that estrogen regulates in the cells of the immune system. Our laboratory has recently shown that *in vivo* estrogen-treated C57BL/6 mice have increased splenic neutrophils comparable to that noticed in female autoimmune-prone MRL/lpr or C57BL/6-lpr (11). Estrogens can also alter macrophage function by regulating chemotaxis, phagocytic activity, and induction of cytokines, iNOS, and nitric oxide (12–16). Estrogen can also enhance differentiation of immature *DCs* into mature functional DCs, and regulate the expression of cytokines and chemokines such as IL-6, IL-10, CXCL8, and CCL2 (17, 18). Overall, multiple studies have demonstrated that estrogens can affect innate immune cell signaling (19–21).

**Table 1:** **List of key selected genes that are regulated by estrogen in cells of innate and adaptive immune system**.

Immune cell	List of genes	Reference
Neutrophil	CINC-1, CINC-2β, CINC-3, TNFα, IL-6, IL-1β	(8–10)
Macrophage	iNOS, NO, IL-6, TNFα	(12–16)
Dendritic cells	IL-6, IL-10, CXCL8, CCL2, TGFβ, IL-23, IL-12	(17, 18, 22)
Th1	IFNγ	(23–25)
Th2	IL-4	(26)
Tregs	FoxP3, PD-1, CTLA-4	(27–30)
B cells	Immunoglobulin, CD22, SHP-1, Bcl-2, VCAM-1	(31)

Estrogen has been shown to modulate all subsets of T cells that include CD4^+^ (Th1, Th2, Th17, and Tregs) and CD8^+^ cells (32–35). Extensive studies have demonstrated that estrogen modulates IFNγ-secreting *Th1 cells* by enhancing IFNγ expression in both human and mice (23–25), which are potentially mediated by direct interaction of ER with Estrogen-response element (ERE) in the promoter region of the *Ifn*γ gene (24) and/or up-regulating Th-1-specific transcription factor T-bet (25, 36). ERα-deficient mice have decreased IFNγ^+^-secreting cells in lymph nodes, suggesting estrogen-driven Th1 cell responsiveness is dependent on ERα-mediated signaling (37). Estrogen’s effect on Th2 cells and its prototypic cytokine, IL-4 is less marked, either having no effect (25, 38, 39) or stimulatory effect of estrogen on IL-4 secretion and *GATA-3 expression* (26) or a positive correlation between menstrual estrogen cycle levels and IL-4 (40). Interestingly, high levels of estrogen (e.g., pregnancy level) are known to skew the immune response from Th1 (IFNγ) to Th2 (IL-4) (41–43). The effects of estrogen on Th17 subset have also been recently reported, albeit with varied response to estrogen depending on the experimental conditions. In periodontal ligament cells culture, addition of estrogen enhances IL-1β-mediated IL-17F production (44). In adult cystic fibrosis male mice, estrogen increases the severity of pneumonia, in part by increased Th17-regulated inflammation (45). However, it has also been shown that estrogen deficiency in postmenopausal women is associated with increased IL-17A levels (46). Estrogen also promotes the expansion and frequency of Treg cells, which play a critical role in downregulating immune responses (28, 30) and upregulating the expression of FoxP3, PD-1, and CTLA-4 via ERα-mediated signaling (27–30). Protective effects of estrogen in autoimmune conditions such as MS and RA are believed to be due to a combined result of estrogen-mediated Treg expansion and activation (27, 47, 48).

Estrogen can also have profound effects on B cell differentiation, activity, function (49, 50), and survival by increasing expression of genes such as *cd22, shp-1, bcl-2*, and *vcam-1* (31) Estrogen has been shown to increase plasma cell and autoantibody producing cells numbers (49, 51). Although signaling by either ERα or β has shown to alter B cell maturation, ERα engagement has been shown to be critical for autoimmunity (52).

The outcome of response of estrogen on the immune system can vary depending upon the level of estrogens, cell type, activation state of cells, local environment, and the experimental context. In many of these studies, it is unclear if estrogenic effects are mediated through ER-dependent or -independent pathways. Nonetheless, the estrogen-mediated effects are apparent in all major innate and adaptive immune cells.

## Estrogen Receptor Expression in the Cells of the Immune System

**Figure 1:**
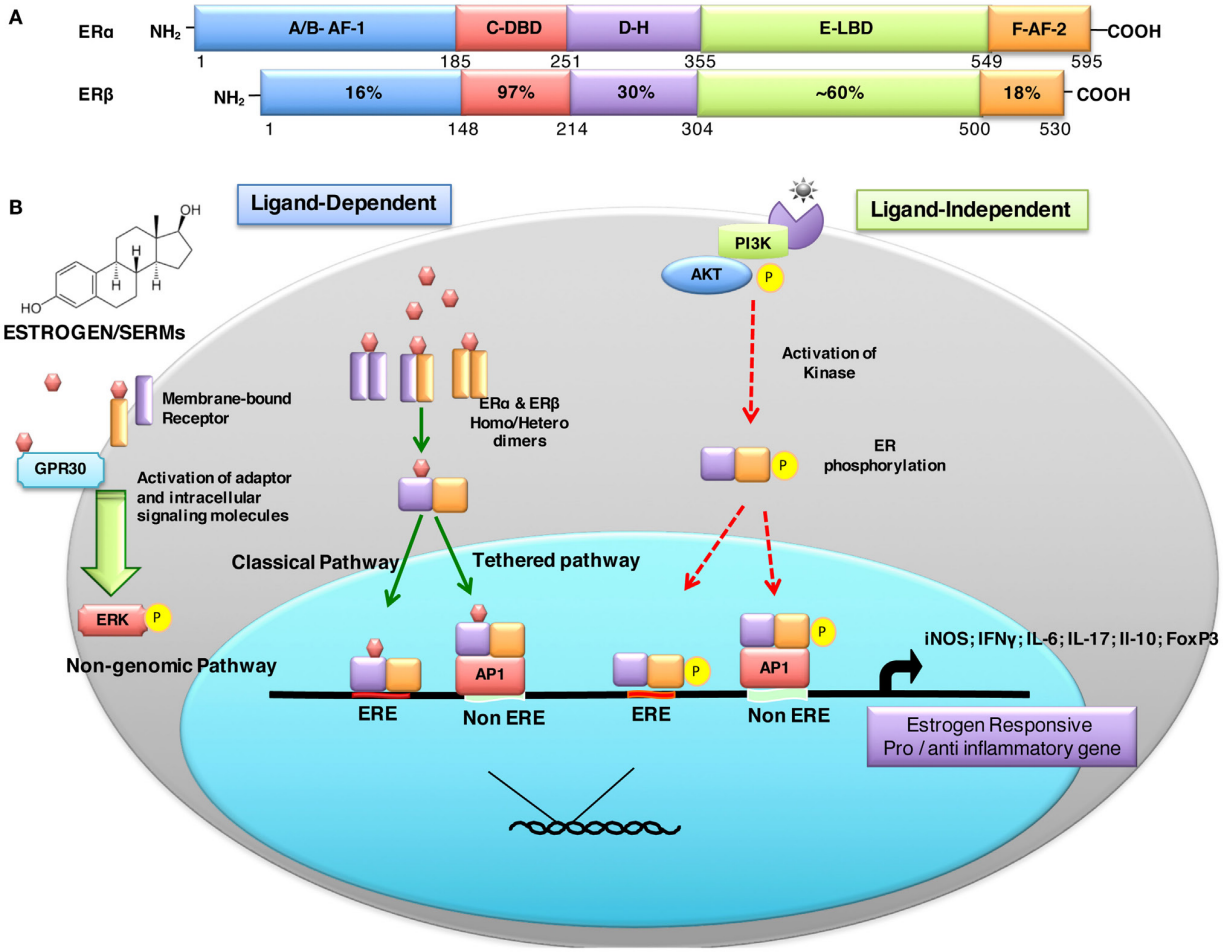
**Structural description and percent sequence homology of human ERα and ERβ and schematic representation of estrogen receptor ligand-dependent and ligand-independent signaling**. **(A)** shows comparison of human estrogen receptor α (595 aa) and a shorter estrogen receptor β (530 aa). These receptors are evolutionarily conserved and have five distinct structural and functional domains: DNA-binding domain (DBD; C domain), hinge domain (D), ligand-binding domain (LBD; E/F domain), and two transcriptional activation function domains AF-1 (in A/B domain) and AF-2 (in F domain). The binding of ligand (estrogens) to E domain results in conformational changes in the receptor (homo/hetero dimerization). The receptor dimer than translocates inside nuclei with the help of D domain. This domain is also important for post-translational modifications of receptor by acetylation, lipophilic moieties, and ubiquitination. The C domain then recognizes and binds to estrogen-response element (ERE) in DNA. AF-2 region interacts with co-regulatory proteins in ligand-dependent pathway. However, AF-1 region is activated in ligand-independent manner (54). **(B)** ER-mediated signaling occurs in a ligand-dependent (green arrows) and ligand-independent (red arrows). The ligand-dependent pathway is triggered by binding of either endogenous hormone or a synthetic compound to the ligand-binding domain of ERs in the cytosol. Different ligands induce unique conformational changes of ERs, and receptor dimerization (homodimers: ERα:ERα or ERβ:ERβ or heterodimer: ERα:ERβ), which then translocate into nuclei and bind to specific EREs (consisting of a 5-bp palindrome with a 3-bp spacer; *GGTCAnnnTGACC*) in the regulatory regions of estrogen responsive genes. This is also called “*classical*” signaling pathway. In “*tethered*” signaling pathway, ligand-activated ERs interact with other transcription factor complexes and bind to non-EREs by attaching to other transcription factors and not with ERE directly. In third ligand-dependent “*non-genomic*” pathway, ligand interacts with plasma membrane-bound ERs via palmitoylation on cysteine447, which results in activation of cytoplasmic signaling pathways, such as protein kinase C (PKC). In *ligand-independent signaling* pathway, there is phosphorylation/activation of ERs by other active signaling cascades in a cell (55). This activation results in both direct ERE and non-ERE dependent genomic actions. Abbreviation: Akt, protein kinase B; AP-1, activator protein 1; ERE, estrogen-response element; ER, estrogen receptor; ERK, extracellular signal-regulated kinase; FoxP3, fork box 3; GRP30, an orphan G-protein coupled receptor 30; iNOS, inducible nitric oxide synthase; PI3K, phosphatidylinositol-4, 5-bisphosphate 3-kinase; P, indicates phosphorylation.

Estrogen-mediated signaling is a result of fine-tuned balance between two distinct receptors ERα (NR3A1) and ERβ (NR3A2) that are encoded by *ESR-1* and *ESR-2* genes expressed on human chromosomes 6 and 14, respectively (53). These receptors act as ligand-activated transcription factors and, therefore, directly regulate a broad range of estrogen-responsive genes. The biochemical similarities and differences between ERα and ERβ are depicted in Figure 1A. Eventhough both ERs have comparable affinity to estrogen and recognize the same ERE, they may have distinct, non-overlapping or even antagonist effects. There are different parameters that determine the overall effect of estrogen receptor-mediated signaling. These factors include: (i) differential distribution and expression of ERs in various cells and tissues, (ii) homo or hetero dimerization of the receptor, (iii) distinct splice variant ER isoforms, (iv) diverse signaling pathways triggered, (v) interaction with specific co-activators/-repressors, (vi) transactivation, (vii) physiological or pathological states, and (viii) local tissue milieu, among others.

In most cells of the immune system ERα is expressed, which includes hematopoietic cells, bone marrow, thymus stromal cells and thymocytes (6, 7, 56–59), and murine splenic DC and peritoneal macrophages (60). Whereas ERβ has a more restricted cellular expression and is preferentially expressed in thymus and spleen of human mid-gestational fetus (61), lymphocytes in human lymph nodes, rat thymocyte and stromal cells (58), murine bone marrow, and thymus (62, 63). Among T lymphocytes, CD4^+^ cells have more ERα levels compared to ERβ whereas CD8^+^ cells have low expression of both receptors. B cells, unlike CD4 cells, have more ERβ than ERα (64). Effect of ERα on the immune system is generally considered to be developmentally more prominent than ERβ (37) since ERα-deficient mice exhibit hypoplastic thymus and spleen (65, 66), increased immature double-positive thymocytes (CD4^+^CD8^+^) and decreased CD4^+^CD8^−^ cells. The balance between ERα and ERβ to maintain a physiological state is underlined by the observations that mice lacking ERα have increased immune complex-mediated glomerulonephritis, proteinuria, infiltration of B cells in kidney, damage of tubular cells, and presence of serum anti-DNA antibodies (66, 67). The natural varied expression of ERs in different tissues and cell types, and during maturity, finely balances the overall outcome of estrogen-mediated immune responses.

## Estrogen Receptor-Mediated Cell Signaling

In large part, estrogen mediates its effects by binding to specific estrogen receptors and triggering distinct signaling pathways to regulate a broad range of estrogen responsive genes. ER-mediated signaling can be broadly classified as either ligand dependent or ligand independent (Figure 1B). Additionally, post-translational modifications of ERs also affect ER signaling and biological functions. These include: phosphorylation stimulates signaling; glycosylation is important for ER localization; acetylation enhances ER-DNA binding activity, hormone sensitivity, and transcriptional activity; sumoylation favors ERα-dependent transcription; nitrosylation reduces DNA binding ability; ubiquitination promotes degradation; myristoylation and palmitoylation affect cross-talk of ERs with membrane proteins, trafficking, as well as signal transduction (68). It is thus not surprising that varied ER-mediated effects in different experimental and clinical situations may in part be due to local differential post-translational modifications of ER.

## Estrogen and Autoimmune Diseases

The pathogenesis of autoimmune diseases remains unclear despite extensive research over a few decades. However, multiple factors that regulate autoimmune diseases have been identified. These include: genetics; epigenetics (miRNA, methylation, and histone deacetylation); infections; and external and internal environmental triggers including hormones and microbiome. A majority of autoimmune diseases occur predominantly in women, a feature also noted in many animal models of autoimmune diseases (5, 69). Initially, it was presumed that the sex bias is related to differences in sex steroids, but it is now evident that several other factors contribute to the sex bias of autoimmune diseases that include X chromosomal abnormalities, X-chromosomal inactivation, and fetal microchimerism. The effects of sex hormones (such as estrogens) on autoimmune diseases cannot be generalized and is context/disease-dependent. It is not surprising that the outcome of estrogen-mediated autoimmune responses is different among autoimmune diseases since estrogens affect all cells of the immune system, and the triggering and pathogenic mechanisms are varied among different diseases. This aspect of differential estrogen-mediated effects in autoimmune diseases, in two classical female-predominant diseases: an organ-specific [multiple sclerosis (MS)] and a non-organ-specific autoimmune disease (SLE), are highlighted below.

### Estrogen and MS

Multiple sclerosis (MS) and its experimental model (EAE) are characterized by the presence of myelin antigens reactive CD4^+^ T cells in the CNS, demyelination of axons resulting in axonal death, and altered CNS function. The women to men ratio for disease prevalence ranges from 2.3 to 3.5:1 (70). The exact mechanism of this female predisposition of MS remains unknown. Yet intriguingly, the female sex hormone estrogen is protective in MS. Estrogens have been shown to have anti-inflammatory and neuroprotective effect in MS and EAE. Estrogen through principally ERα-dependent mechanism decreases autoantigen-specific proinflammatory biomolecules (such as IFNγ, TNFα, IL-17, iNOS, and MCP-1), and inhibits inflammation and demyelination (32, 47, 48, 71). ERβ agonist diarylpropionitrile (DPN) protects oligodendrocytes by increasing endogenous myelination (72). A recent report has demonstrated that estrogen protects gray matter atrophy in EAE (73). In addition in the EAE model, estrogen inhibits CD4^+^ T cells expansion, increases proportions of Tregs and CD4^+^CD8^−^ suppressor T cells (74), increases T cell apoptosis (75), and markedly alters expression pattern of 315 genes in spinal cord tissue of mice protected from EAE (76). In pregnant EAE mice, there is reduced CNS pathology and decreased TNFα and IL-17 production when compared to non-pregnant controls (77). The rate of relapse in females increases postpartum at a phase when there is a marked decrease in estrogen levels when compared to pregnancy levels (78, 79)*. In vivo* estriol treatment promotes generation of tolerogenic DCs with increased activation markers (CD80 and CD86), inhibitory costimulatory markers (PD-L1, PD-L2, B7-H3, and B7-H4), and increased anti-inflammatory (IL-10 and TGFβ) and decreased pro-inflammatory (IL-12, IL-23, and IL-6) cytokine mRNA expression (22).

### Estrogen and SLE

The female:male susceptibility ratio for SLE is 9–20:1 (69, 80–82). Although the precise effect of estrogens in human SLE is not clear, unlike MS, a majority of studies have shown that estrogen is not protective in SLE. Rather, studies in a number of relevant animal models for SLE show that estrogen may have opposite effects. Several studies have shown that estrogen enhances severity and flares of disease in both human and animal models (83, 84). Estrogen enhances anti-double-stranded DNA antibody and IgG, IgM production by PBMCs, and serum from patients with SLE (85). Estrogen increases reactivity to exogenous antigens and also increases expression of endogenous autoantigens, e.g., human endogenous retrovirus (HERV) (86, 87), which molecularly mimics RNP antigens, and is increased in SLE patients (88). Estrogen also promotes systemic inflammation and induction of B cell activating factor and IFN signature genes (89). Over 50% of the genes that are altered during menstrual cycle are also markedly altered in SLE patients when compared to healthy controls (90). Tumor necrosis factor receptor superfamily member (TNFRSF14) also called Herpes virus entry mediator (HVEM), which interacts with B and T lymphocyte attenuator (BTLA) and downregulates lymphocyte activation and homeostasis (91), is altered in normal females when compared to males, as well as in SLE patients when compared to normal controls (90). Although during a menstrual cycle, there is estrogen-mediated increased expression of TNFRSF14 mRNA in PBMCs, in SLE patients, there is decreased TNFRSF14 mRNA, which results in only partial immune suppression by BTLA culminating in overall immune enhancement (91, 92).

Altered ER expression in SLE patients and different murine lupus models potentially results in their hyper-reactivity to estrogen (93, 94). There is increased ERα and decreased ERβ mRNA expression in PBMCs of SLE patients (94). Increased ERα expressing CD4^+^ and CD8^+^ T cells and ERα^+^ DCs and macrophages in estrogen-treated autoimmune SNF_(1)_ mice as compared to control DBF_(1)_ mice has also been reported (95). It is evident from different studies that ERα-mediated signaling is required for exaggeration of lupus disease (95, 96). Furthermore, ERα polymorphism is also reported in SLE patients (97, 98). Different risk alleles susceptibility loci, lupus-susceptibility genes, and SNPs have also been identified in SLE patients such as interferon regulatory factor (IRF5); HLA-DR2 and HLA-DR3, Ifi202 of Ifi200-family, PTPN22, CTLA4, STAT4 and BANK1, TLR 7, 8, 9, etc. (99–103). Nuclear antigens activate TLR7- and TLR9-mediated IRF5 activation, which increases IFNα secretion in SLE patients (104, 105). Estrogen signaling via ERα also upregulates IRF5 mRNA (106). Recent reports have implied an important role for TLR8 and TLR9 in checking TLR7-mediated spontaneous autoimmunity in mice (107). In addition, X-linked TLR8 dosage also plays critical role in increased susceptibility of females to SLE (108). Single copy of TLR8 in 564Igi *Tlr7/9^−/−^* mice is not enough for autoantibody production, granulopoeisis, and *Ifn-I* expression (108). Although unclear how estrogen promotes lupus in relevant EAE, it is conceivable that estrogen may have complex multicellular effects that include altered ER signaling, stimulating pro-inflammatory cytokines from Th and other cells, augmenting pathogenic autoantibodies by favoring localized action of Th2 cells, aberrant TLR-mediated signaling, enhancing autoantigen presentation, downregulating regulatory apparatus, and dysregulating microRNA expression.

## Conclusion

There is now a wealth of data that affirms estrogen regulates various facets of the immune system via complex molecular mechanisms. Although both MS and SLE are autoimmune diseases, they differ in many respects that include target pathological organs, triggering and pathogenic mechanisms, predominant effector cell type(s), genetics, and epigenetics among others. Given that estrogen affects all cells of the immune system as well as non-lymphoid tissues (e.g., vascular endothelial cells) that are in proximity of target tissues, it is conceivable that estrogen will have different local effects. Therefore, it is not surprising that estrogen has been shown to have different effects in various autoimmune diseases. While sex hormones may play a role in sex-differences in autoimmune diseases, clearly sex hormones alone do not exclusively contribute to this sex differential susceptibility. Precisely why females are more susceptible to autoimmune diseases continues to be an intriguing area of investigation. It is therefore important to fully understand the complex interaction of estrogen in context-specific situations. It is plausible that estrogen may have varied epigenetic effects (microRNAs, histone modifications, and/or acetylation) (109) and different ER-mediated post-translational modifications in different diseases such as in MS and SLE. Indeed, we have shown that estrogen induces signature miRNA expression in lymphocytes (14) and accelerates the expression of lupus-associated miRNAs in lupus-prone mice (110). It is also likely that the microbiome in MS and SLE are different. Microbiome can affect sex hormone production and vice versa (111, 112), which in turn affects systemic immune responses. Local estrogen and response levels altered by microbiome may be different in these two autoimmune diseases, an aspect that merits investigations. It is conceivable that any immune cell (innate and adaptive) that expresses ERα and/or ERβ can potentially respond to estrogen in a context-dependent fashion, which will affect the outcome of immune or autoimmune responses. Given spatial and temporal expression of ERs, it is important to have a comprehensive knowledge and evaluation of ER expression in a particular tissue before designing potential ER-targeted therapies. In relation to personalized medicine, with the advent of highly sensitive molecular arrays, metagenomics and bioinformatics, it is plausible to integrate these techniques to better predict the estrogen-mediated immunomodulatory effects in a disease-specific fashion.

## Author Contributions

DK and SA designed the work, drafted and revised the work, and finally approved the version to be published and agree to be accountable for all aspects of the work.

## Conflict of Interest Statement

The authors declare that the research was conducted in the absence of any commercial or financial relationships that could be construed as a potential conflict of interest.

## References

[1] Smith EP Boyd J Frank GR Takahashi H Cohen RM Specker B Estrogen resistance caused by a mutation in the estrogen-receptor gene in a man. N Engl J Med (1994) 331(16):1056–61. 10.1056/NEJM199410203311604 8090165

[2] Carani C Qin K Simoni M Faustini-Fustini M Serpente S Boyd J Effect of testosterone and estradiol in a man with aromatase deficiency. N Engl J Med (1997) 337(2):91–5. 10.1056/NEJM199707103370204 9211678

[3] Gustafsson JA. What pharmacologists can learn from recent advances in estrogen signalling. Trends Pharmacol Sci (2003) 24(9):479–85. 10.1016/S0165-6147(03)00229-3 12967773

[4] Ansar Ahmed S Karpuzoglu E Khan D Effect of sex steroids on innate and adaptive immunity. In: Klein SL Roberts CW, editors. Sex Hormones and Immunity to Infection. London: Springer (2010). p. 19–51.

[5] Khan D Cowan C Ansar Ahmed S Estrogen and signaling in the cells of immune system. Adv Neuroimm Biol (2012) 3(1):73–93. 10.3233/NIB-2012-012039

[6] Kovats S. Estrogen receptors regulate an inflammatory pathway of dendritic cell differentiation: mechanisms and implications for immunity. Horm Behav (2012) 62(3):254–62. 10.1016/j.yhbeh.2012.04.011 22561458 PMC3415586

[7] Kovats S. Estrogen receptors regulate innate immune cells and signaling pathways. Cell Immunol (2015) 294(2):63–9. 10.1016/j.cellimm.2015.01.018 25682174 PMC4380804

[8] Hsu JT Kan WH Hsieh CH Choudhry MA Schwacha MG Bland KI Mechanism of estrogen-mediated attenuation of hepatic injury following trauma-hemorrhage: Akt-dependent HO-1 up-regulation. J Leukoc Biol (2007) 82(4):1019–26. 10.1189/jlb.0607355 17656650

[9] Yu HP Hsieh YC Suzuki T Choudhry MA Schwacha MG Bland KI Mechanism of the nongenomic effects of estrogen on intestinal myeloperoxidase activity following trauma-hemorrhage: up-regulation of the PI-3K/Akt pathway. J Leukoc Biol (2007) 82(3):774–80. 10.1189/jlb.0307182 17586659

[10] Cuzzocrea S Genovese T Mazzon E Esposito E Di Paola R Muia C Effect of 17beta-estradiol on signal transduction pathways and secondary damage in experimental spinal cord trauma. Shock (2008) 29(3):362–71. 10.1097/SHK.0b013e31814545dc 17704735

[11] Cowan C Dai R Heid B Ansar Ahmed S Phenotypic and functional characterization of neutrophils from lupus-prone mice. American Association of Immunologists Annual Meeting, Immunology 2014. Pittsburg, PA (2014).

[12] Cutolo M Sulli A Capellino S Villaggio B Montagna P Seriolo B Sex hormones influence on the immune system: basic and clinical aspects in autoimmunity. Lupus (2004) 13(9):635–8. 10.1191/0961203304lu1094oa 15485092

[13] Karpuzoglu E Ansar Ahmed S. Estrogen regulation of nitric oxide and inducible nitric oxide synthase (iNOS) in immune cells: implications for immunity, autoimmune diseases, and apoptosis. Nitric Oxide (2006) 15(3):177–86. 10.1016/j.niox.2006.03.009 16647869

[14] Dai R Phillips RA Zhang Y Khan D Crasta O Ansar Ahmed S. Suppression of LPS-induced Interferon-gamma and nitric oxide in splenic lymphocytes by select estrogen-regulated microRNAs: a novel mechanism of immune modulation. Blood (2008) 112(12):4591–7. 10.1182/blood-2007-10-116376 18791161 PMC2597130

[15] Hsieh CH Nickel EA Chen J Schwacha MG Choudhry MA Bland KI Mechanism of the salutary effects of estrogen on kupffer cell phagocytic capacity following trauma-hemorrhage: pivotal role of Akt activation. J Immunol (2009) 182(7):4406–14. 10.4049/jimmunol.0803423 19299741 PMC2814125

[16] Karpuzoglu E Phillips RA Dai R Graniello C Gogal RM Jr Ansar Ahmed S. Signal transducer and activation of transcription (STAT) 4beta, a shorter isoform of interleukin-12-induced STAT4, is preferentially activated by estrogen. Endocrinology (2009) 150(3):1310–20. 10.1210/en.2008-0832 18988675 PMC2654738

[17] Liu HY Buenafe AC Matejuk A Ito A Zamora A Dwyer J Estrogen inhibition of EAE involves effects on dendritic cell function. J Neurosci Res (2002) 70(2):238–48. 10.1002/jnr.10454 12271473

[18] Bachy V Williams DJ Ibrahim MA. Altered dendritic cell function in normal pregnancy. J Reprod Immunol (2008) 78(1):11–21. 10.1016/j.jri.2007.09.004 18006075

[19] Cunningham MA Wirth JR Naga O Eudaly J Gilkeson GS. Estrogen receptor alpha binding to ERE is required for full Tlr7- and Tlr9-induced inflammation. SOJ Immunol (2014) 2(1):7. 10.15226/soji.2014.00107 25061615 PMC4106444

[20] Laffont S Rouquie N Azar P Seillet C Plumas J Aspord C X-Chromosome complement and estrogen receptor signaling independently contribute to the enhanced TLR7-mediated IFN-alpha production of plasmacytoid dendritic cells from women. J Immunol (2014) 193(11):5444–52. 10.4049/jimmunol.1303400 25339659

[21] Stojic-Vukanic Z Nacka-Aleksic M Bufan B Pilipovic I Arsenovic-Ranin N Djikic J 17beta-Estradiol influences in vitro response of aged rat splenic conventional dendritic cells to TLR4 and TLR7/8 agonists in an agonist specific manner. Int Immunopharmacol (2015) 24(1):24–35. 10.1016/j.intimp.2014.11.008 25479725

[22] Papenfuss TL Powell ND McClain MA Bedarf A Singh A Gienapp IE Estriol generates tolerogenic dendritic cells in vivo that protect against autoimmunity. J Immunol (2011) 186(6):3346–55. 10.4049/jimmunol.1001322 21317386 PMC3600583

[23] Grasso G Muscettola M The influence of beta-estradiol and progesterone on interferon gamma production in vitro. Int J Neurosci (1990) 51(3–4):315–7. 10.3109/00207459008999730 2126259

[24] Fox HS Bond BL Parslow TG. Estrogen regulates the IFN-gamma promoter. J Immunol (1991) 146(12):4362–7. 1904081

[25] Karpuzoglu-Sahin E Hissong BD Ansar Ahmed S. Interferon-gamma levels are upregulated by 17-beta-estradiol and diethylstilbestrol. J Reprod Immunol (2001) 52(1–2):113–27. 10.1016/S0165-0378(01)00117-6 11600182

[26] Lambert KC Curran EM Judy BM Milligan GN Lubahn DB Estes DM. Estrogen receptor alpha (ERalpha) deficiency in macrophages results in increased stimulation of CD4+ T cells while 17beta-estradiol acts through ERalpha to increase IL-4 and GATA-3 expression in CD4+ T cells independent of antigen presentation. J Immunol (2005) 175(9):5716–23. 10.4049/jimmunol.175.9.5716 16237062

[27] Polanczyk MJ Carson BD Subramanian S Afentoulis M Vandenbark AA Ziegler SF Cutting edge: estrogen drives expansion of the CD4+CD25+ regulatory T cell compartment. J Immunol (2004) 173(4):2227–30. 10.4049/jimmunol.173.4.2227 15294932

[28] Polanczyk MJ Hopke C Huan J Vandenbark AA Offner H. Enhanced FoxP3 expression and Treg cell function in pregnant and estrogen-treated mice. J Neuroimmunol (2005) 170(1–2):85–92. 10.1016/j.jneuroim.2005.08.023 16253347

[29] Polanczyk MJ Hopke C Vandenbark AA Offner H. Treg suppressive activity involves estrogen-dependent expression of programmed death-1 (PD-1). Int Immunol (2007) 19(3):337–43. 10.1093/intimm/dxl151 17267414

[30] Tai P Wang J Jin H Song X Yan J Kang Y Induction of regulatory T cells by physiological level estrogen. J Cell Physiol (2008) 214(2):456–64. 10.1002/jcp.21221 17654501

[31] Grimaldi CM Cleary J Dagtas AS Moussai D Diamond B. Estrogen alters thresholds for B cell apoptosis and activation. J Clin Invest (2002) 109(12):1625–33. 10.1172/JCI0214873 12070310 PMC151010

[32] Lelu K Laffont S Delpy L Paulet PE Perinat T Tschanz SA Estrogen receptor alpha signaling in T lymphocytes is required for estradiol-mediated inhibition of Th1 and Th17 cell differentiation and protection against experimental autoimmune encephalomyelitis. J Immunol (2011) 187(5):2386–93. 10.4049/jimmunol.1101578 21810607

[33] Priyanka HP Krishnan HC Singh RV Hima L Thyagarajan S. Estrogen modulates in vitro T cell responses in a concentration- and receptor-dependent manner: effects on intracellular molecular targets and antioxidant enzymes. Mol Immunol (2013) 56(4):328–39. 10.1016/j.molimm.2013.05.226 23911387

[34] Robinson DP Hall OJ Nilles TL Bream JH Klein SL 17beta-estradiol protects females against influenza by recruiting neutrophils and increasing virus-specific CD8 T cell responses in the lungs. J Virol (2014) 88(9):4711–20. 10.1128/JVI.03609-13 24522912 PMC3993800

[35] Karpuzoglu-Sahin E Zhi-Jun Y Lengi A Sriranganathan N Ansar Ahmed S. Effects of long-term estrogen treatment on IFN-gamma, IL-2 and IL-4 gene expression and protein synthesis in spleen and thymus of normal C57BL/6 mice. Cytokine (2001) 14(4):208–17. 10.1006/cyto.2001.0876 11448120

[36] Karpuzoglu E Phillips RA Gogal RM Jr Ansar Ahmed S. IFN-gamma-inducing transcription factor, T-bet is upregulated by estrogen in murine splenocytes: role of IL-27 but not IL-12. Mol Immunol (2007) 44(7):1819–25. 10.1016/j.molimm.2006.08.005 17046061 PMC3097111

[37] Maret A Coudert JD Garidou L Foucras G Gourdy P Krust A Estradiol enhances primary antigen-specific CD4 T cell responses and Th1 development in vivo. Essential role of estrogen receptor alpha expression in hematopoietic cells. Eur J Immunol (2003) 33(2):512–21. 10.1002/immu.200310027 12645950

[38] Matejuk A Adlard K Zamora A Silverman M Vandenbark AA Offner H. 17 beta-estradiol inhibits cytokine, chemokine, and chemokine receptor mRNA expression in the central nervous system of female mice with experimental autoimmune encephalomyelitis. J Neurosci Res (2001) 65(6):529–42. 10.1002/jnr.1183 11550221

[39] Sakazaki F Ueno H Nakamuro K. 17beta-Estradiol enhances expression of inflammatory cytokines and inducible nitric oxide synthase in mouse contact hypersensitivity. Int Immunopharmacol (2008) 8(5):654–60. 10.1016/j.intimp.2008.01.007 18387507

[40] Verthelyi D Klinman DM. Sex hormone levels correlate with the activity of cytokine-secreting cells in vivo. Immunology (2000) 100(3):384–90. 10.1046/j.1365-2567.2000.00047.x 10929062 PMC2327016

[41] Sabahi F Rola-Plesczcynski M O’Connell S Frenkel LD. Qualitative and quantitative analysis of T lymphocytes during normal human pregnancy. Am J Reprod Immunol (1995) 33(5):381–93. 10.1111/j.1600-0897.1995.tb00907.x 7576120

[42] Marzi M Vigano A Trabattoni D Villa ML Salvaggio A Clerici E Characterization of type 1 and type 2 cytokine production profile in physiologic and pathologic human pregnancy. Clin Exp Immunol (1996) 106(1):127–33. 10.1046/j.1365-2249.1996.d01-809.x 8870710 PMC2200555

[43] Matalka KZ. The effect of estradiol, but not progesterone, on the production of cytokines in stimulated whole blood, is concentration-dependent. Neuro Endocrinol Lett (2003) 24(3–4):185–91. 14523355

[44] Konermann A Winter J Novak N Allam JP Jager A. Verification of IL-17A and IL-17F in oral tissues and modulation of their expression pattern by steroid hormones. Cell Immunol (2013) 285(1–2):133–40. 10.1016/j.cellimm.2013.10.004 24185279

[45] Wang Y Cela E Gagnon S Sweezey NB. Estrogen aggravates inflammation in *Pseudomonas aeruginosa* pneumonia in cystic fibrosis mice. Respir Res (2010) 11:166. 10.1186/1465-9921-11-166 21118573 PMC3006363

[46] Molnar I Bohaty I Somogyine-Vari E. High prevalence of increased interleukin-17A serum levels in postmenopausal estrogen deficiency. Menopause (2014) 21(7):749–52. 10.1097/GME.0000000000000125 24253487

[47] Polanczyk M Zamora A Subramanian S Matejuk A Hess DL Blankenhorn EP The protective effect of 17beta-estradiol on experimental autoimmune encephalomyelitis is mediated through estrogen receptor-alpha. Am J Pathol (2003) 163(4):1599–605. 10.1016/S0002-9440(10)63516-X 14507666 PMC1868300

[48] Offner H Polanczyk M. A potential role for estrogen in experimental autoimmune encephalomyelitis and multiple sclerosis. Ann N Y Acad Sci (2006) 1089:343–72. 10.1196/annals.1386.021 17261780

[49] Verthelyi DI Ansar Ahmed S. Estrogen increases the number of plasma cells and enhances their autoantibody production in nonautoimmune C57BL/6 mice. Cell Immunol (1998) 189(2):125–34. 10.1006/cimm.1998.1372 9790726

[50] Grimaldi CM Jeganathan V Diamond B. Hormonal regulation of B cell development: 17 beta-estradiol impairs negative selection of high-affinity DNA-reactive B cells at more than one developmental checkpoint. J Immunol (2006) 176(5):2703–10. 10.4049/jimmunol.176.5.2703 16493025

[51] Bernardi AI Andersson A Grahnemo L Nurkkala-Karlsson M Ohlsson C Carlsten H Effects of lasofoxifene and bazedoxifene on B cell development and function. Immun Inflamm Dis (2014) 2(4):214–25. 10.1002/iid3.37 25866629 PMC4386916

[52] Hill L Jeganathan V Chinnasamy P Grimaldi C Diamond B Differential roles of estrogen receptors alpha and beta in control of B-cell maturation and selection. Mol Med (2011) 17(3–4):211–20. 10.2119/molmed.2010.00172 21107497 PMC3060981

[53] Thomas C Gustafsson JA. The different roles of ER subtypes in cancer biology and therapy. Nat Rev Cancer (2011) 11(8):597–608. 10.1038/nrc3093-c2 21779010

[54] Krege JH Hodgin JB Couse JF Enmark E Warner M Mahler JF Generation and reproductive phenotypes of mice lacking estrogen receptor beta. Proc Natl Acad Sci USA (1998) 95(26):15677–82. 10.1073/pnas.95.26.15677 9861029 PMC28103

[55] Kato S Endoh H Masuhiro Y Kitamoto T Uchiyama S Sasaki H Activation of the estrogen receptor through phosphorylation by mitogen-activated protein kinase. Science (1995) 270(5241):1491–4. 10.1126/science.270.5241.1491 7491495

[56] Kawashima I Seiki K Sakabe K Ihara S Akatsuka A Katsumata Y. Localization of estrogen receptors and estrogen receptor-mRNA in female mouse thymus. Thymus (1992) 20(2):115–21. 1519316

[57] Seiki K Sakabe K. Sex hormones and the thymus in relation to thymocyte proliferation and maturation. Arch Histol Cytol (1997) 60(1):29–38. 10.1679/aohc.60.29 9161687

[58] Mor G Munoz A Redlinger R Jr Silva I Song J Lim C The role of the Fas/Fas ligand system in estrogen-induced thymic alteration. Am J Reprod Immunol (2001) 46(4):298–307. 10.1034/j.1600-0897.2001.d01-16.x 11642679

[59] Carreras E Turner S Paharkova-Vatchkova V Mao A Dascher C Kovats S. Estradiol acts directly on bone marrow myeloid progenitors to differentially regulate GM-CSF or Flt3 ligand-mediated dendritic cell differentiation. J Immunol (2008) 180(2):727–38. 10.4049/jimmunol.180.2.727 18178810

[60] Lambert KC Curran EM Judy BM Lubahn DB Estes DM. Estrogen receptor-alpha deficiency promotes increased TNF-alpha secretion and bacterial killing by murine macrophages in response to microbial stimuli in vitro. J Leukoc Biol (2004) 75(6):1166–72. 10.1189/jlb.1103589 15020652

[61] Brandenberger AW Tee MK Lee JY Chao V Jaffe RB. Tissue distribution of estrogen receptors alpha (ER-alpha) and beta (ER-beta) mRNA in the midgestational human fetus. J Clin Endocrinol Metab (1997) 82(10):3509–12. 10.1210/jcem.82.10.4400 9329394

[62] Smithson G Couse JF Lubahn DB Korach KS Kincade PW. The role of estrogen receptors and androgen receptors in sex steroid regulation of B lymphopoiesis. J Immunol (1998) 161(1):27–34. 9647203

[63] Vidal O Kindblom LG Ohlsson C. Expression and localization of estrogen receptor-beta in murine and human bone. J Bone Miner Res (1999) 14(6):923–9. 10.1359/jbmr.1999.14.6.923 10352100

[64] Phiel KL Henderson RA Adelman SJ Elloso MM. Differential estrogen receptor gene expression in human peripheral blood mononuclear cell populations. Immunol Lett (2005) 97(1):107–13. 10.1016/j.imlet.2004.10.007 15626482

[65] Staples JE Gasiewicz TA Fiore NC Lubahn DB Korach KS Silverstone AE. Estrogen receptor alpha is necessary in thymic development and estradiol-induced thymic alterations. J Immunol (1999) 163(8):4168–74. 10510352

[66] Erlandsson MC Ohlsson C Gustafsson JA Carlsten H. Role of oestrogen receptors alpha and beta in immune organ development and in oestrogen-mediated effects on thymus. Immunology (2001) 103(1):17–25. 10.1046/j.1365-2567.2001.01212.x 11380688 PMC1783216

[67] Shim GJ Kis LL Warner M Gustafsson JA. Autoimmune glomerulonephritis with spontaneous formation of splenic germinal centers in mice lacking the estrogen receptor alpha gene. Proc Natl Acad Sci USA (2004) 101(6):1720–4. 10.1073/pnas.0405099101 14745006 PMC341834

[68] Ascenzi P Bocedi A Marino M. Structure-function relationship of estrogen receptor alpha and beta: impact on human health. Mol Aspects Med (2006) 27(4):299–402. 10.1016/j.mam.2006.07.001 16914190

[69] Ansar Ahmed S Penhale WJ Talal N. Sex hormones, immune responses, and autoimmune diseases. Mechanisms of sex hormone action. Am J Pathol (1985) 121(3):531–51. 3907369 PMC1887926

[70] Harbo HF Gold R Tintore M. Sex and gender issues in multiple sclerosis. Ther Adv Neurol Disord (2013) 6(4):237–48. 10.1177/1756285613488434 23858327 PMC3707353

[71] Spence RD Wisdom AJ Cao Y Hill HM Mongerson CR Stapornkul B Estrogen mediates neuroprotection and anti-inflammatory effects during EAE through ERalpha signaling on astrocytes but not through ERbeta signaling on astrocytes or neurons. J Neurosci (2013) 33(26):10924–33. 10.1523/JNEUROSCI.0886-13.2013 23804112 PMC3693061

[72] Khalaj AJ Yoon J Nakai J Winchester Z Moore SM Yoo T Estrogen receptor (ER) beta expression in oligodendrocytes is required for attenuation of clinical disease by an ERbeta ligand. Proc Natl Acad Sci U S A (2013) 110(47):19125–30. 10.1073/pnas.1311763110 24191028 PMC3839759

[73] MacKenzie-Graham AJ Rinek GA Avedisian A Morales LB Umeda E Boulat B Estrogen treatment prevents gray matter atrophy in experimental autoimmune encephalomyelitis. J Neurosci Res (2012) 90(7):1310–23. 10.1002/jnr.23019 22411609 PMC3350614

[74] Pettersson A Ciumas C Chirsky V Link H Huang YM Xiao BG. Dendritic cells exposed to estrogen in vitro exhibit therapeutic effects in ongoing experimental allergic encephalomyelitis. J Neuroimmunol (2004) 156(1–2):58–65. 10.1016/j.jneuroim.2004.07.004 15465596

[75] Xiao BG Liu X Link H. Antigen-specific T cell functions are suppressed over the estrogen-dendritic cell-indoleamine 2,3-dioxygenase axis. Steroids (2004) 69(10):653–9. 10.1016/j.steroids.2004.05.019 15465110

[76] Matejuk A Dwyer J Hopke C Vandenbark AA Offner H. 17Beta-estradiol treatment profoundly down-regulates gene expression in spinal cord tissue in mice protected from experimental autoimmune encephalomyelitis. Arch Immunol Ther Exp (Warsz) (2003) 51(3):185–93. 12894873

[77] Gatson NN Williams JL Powell ND McClain MA Hennon TR Robbins PD Induction of pregnancy during established EAE halts progression of CNS autoimmune injury via pregnancy-specific serum factors. J Neuroimmunol (2011) 230(1–2):105–13. 10.1016/j.jneuroim.2010.09.010 20950868 PMC3021646

[78] Confavreux C Hutchinson M Hours MM Cortinovis-Tourniaire P Moreau T. Rate of pregnancy-related relapse in multiple sclerosis. Pregnancy in Multiple Sclerosis Group. N Engl J Med (1998) 339(5):285–91. 10.1056/NEJM199807303390501 9682040

[79] Vukusic S Hutchinson M Hours M Moreau T Cortinovis-Tourniaire P Adeleine P Pregnancy and multiple sclerosis (the PRIMS study): clinical predictors of post-partum relapse. Brain (2004) 127(Pt 6):1353–60. 10.1093/brain/awh152 15130950

[80] Beeson PB. Age and sex associations of 40 autoimmune diseases. Am J Med (1994) 96(5):457–62. 10.1016/0002-9343(94)90173-2 8192178

[81] Lockshin MD. Sex ratio and rheumatic disease: excerpts from an Institute of Medicine report. Lupus (2002) 11(10):662–6. 10.1191/0961203302lu274oa 12413063

[82] Yacoub Wasef SZ. Gender differences in systemic lupus erythematosus. Gend Med (2004) 1(1):12–7. 10.1016/S1550-8579(04)80006-8 16115579

[83] Straub RH. The complex role of estrogens in inflammation. Endocr Rev (2007) 28(5):521–74. 10.1210/er.2007-0001 17640948

[84] Kassi E Moutsatsou P. Estrogen receptor signaling and its relationship to cytokines in systemic lupus erythematosus. J Biomed Biotechnol (2010) 2010:317452. 10.1155/2010/317452 20617147 PMC2896666

[85] Kanda N Tamaki K. Estrogen enhances immunoglobulin production by human PBMCs. J Allergy Clin Immunol (1999) 103(2 Pt 1):282–8. 10.1016/S0091-6749(99)70503-8 9949320

[86] Durali D de Goer de Herve MG Giron-Michel J Azzarone B Delfraissy JF Taoufik Y. In human B cells, IL-12 triggers a cascade of molecular events similar to Th1 commitment. Blood (2003) 102(12):4084–9. 10.1182/blood-2003-02-0518 12893768

[87] Sekigawa I Naito T Hira K Mitsuishi K Ogasawara H Hashimoto H Possible mechanisms of gender bias in SLE: a new hypothesis involving a comparison of SLE with atopy. Lupus (2004) 13(4):217–22. 10.1191/0961203304lu1012ed 15176655

[88] Perl A Nagy G Koncz A Gergely P Fernandez D Doherty E Molecular mimicry and immunomodulation by the HRES-1 endogenous retrovirus in SLE. Autoimmunity (2008) 41(4):287–97. 10.1080/08916930802024764 18432409 PMC5294745

[89] Venkatesh J Yoshifuji H Kawabata D Chinnasamy P Stanevsky A Grimaldi CM Antigen is required for maturation and activation of pathogenic anti-DNA antibodies and systemic inflammation. J Immunol (2011) 186(9):5304–12. 10.4049/jimmunol.1000224 21444762 PMC3192436

[90] Kawasaki M Sekigawa I Nozawa K Kaneko H Takasaki Y Takamori K Changes in the gene expression of peripheral blood mononuclear cells during the menstrual cycle of females is associated with a gender bias in the incidence of systemic lupus erythematosus. Clin Exp Rheumatol (2009) 27(2):260–6. 19473566

[91] Sedy JR Gavrieli M Potter KG Hurchla MA Lindsley RC Hildner K B and T lymphocyte attenuator regulates T cell activation through interaction with herpesvirus entry mediator. Nat Immunol (2005) 6(1):90–8. 10.1038/ni1144 15568026

[92] Cai G Freeman GJ. The CD160, BTLA, LIGHT/HVEM pathway: a bidirectional switch regulating T-cell activation. Immunol Rev (2009) 229(1):244–58. 10.1111/j.1600-065X.2009.00783.x 19426226

[93] Greenstein B Roa R Dhaher Y Nunn E Greenstein A Khamashta M Estrogen and progesterone receptors in murine models of systemic lupus erythematosus. Int Immunopharmacol (2001) 1(6):1025–35. 10.1016/S1567-5769(01)00034-0 11407299

[94] Inui A Ogasawara H Naito T Sekigawa I Takasaki Y Hayashida Y Estrogen receptor expression by peripheral blood mononuclear cells of patients with systemic lupus erythematosus. Clin Rheumatol (2007) 26(10):1675–8. 10.1007/s10067-007-0568-3 17874259

[95] Feng F Nyland J Banyai M Tatum A Silverstone AE Gavalchin J. The induction of the lupus phenotype by estrogen is via an estrogen receptor-alpha-dependent pathway. Clin Immunol (2010) 134(2):226–36. 10.1016/j.clim.2009.10.004 19926524

[96] Li J McMurray RW. Effects of estrogen receptor subtype-selective agonists on autoimmune disease in lupus-prone NZB/NZW F1 mouse model. Clin Immunol (2007) 123(2):219–26. 10.1016/j.clim.2007.03.471 17336162

[97] Lee YJ Shin KS Kang SW Lee CK Yoo B Cha HS Association of the oestrogen receptor alpha gene polymorphisms with disease onset in systemic lupus erythematosus. Ann Rheum Dis (2004) 63(10):1244–9. 10.1136/ard.2003.012583 15361380 PMC1754755

[98] Johansson M Arlestig L Moller B Smedby T Rantapaa-Dahlqvist S. Oestrogen receptor {alpha} gene polymorphisms in systemic lupus erythematosus. Ann Rheum Dis (2005) 64(11):1611–7. 10.1136/ard.2004.032425 15817658 PMC1755265

[99] Choubey D Panchanathan R. Interferon-inducible Ifi200-family genes in systemic lupus erythematosus. Immunol Lett (2008) 119(1–2):32–41. 10.1016/j.imlet.2008.06.001 18598717 PMC2585765

[100] Koga M Kawasaki A Ito I Furuya T Ohashi J Kyogoku C Cumulative association of eight susceptibility genes with systemic lupus erythematosus in a Japanese female population. J Hum Genet (2011) 56(7):503–7. 10.1038/jhg.2011.49 21562514

[101] Chung SA Brown EE Williams AH Ramos PS Berthier CC Bhangale T Lupus nephritis susceptibility loci in women with systemic lupus erythematosus. J Am Soc Nephrol (2014) 25(12):2859–70. 10.1681/ASN.2013050446 24925725 PMC4243339

[102] De Azevêdo Silva J Addobbati C Sandrin-Garcia P Crovella S. Systemic lupus erythematosus: old and new susceptibility genes versus clinical manifestations. Curr Genomics (2014) 15(1):52–65. 10.2174/138920291501140306113715 24653663 PMC3958959

[103] Enevold C Nielsen CH Jacobsen RS Hermansen ML Molbo D Avlund K Single nucleotide polymorphisms in genes encoding toll-like receptors 7, 8 and 9 in Danish patients with systemic lupus erythematosus. Mol Biol Rep (2014) 41(9):5755–63. 10.1007/s11033-014-3447-4 24919757

[104] Salloum R Niewold TB. Interferon regulatory factors in human lupus pathogenesis. Transl Res (2011) 157(6):326–31. 10.1016/j.trsl.2011.01.006 21575916 PMC3096827

[105] Tada Y Kondo S Aoki S Koarada S Inoue H Suematsu R Interferon regulatory factor 5 is critical for the development of lupus in MRL/lpr mice. Arthritis Rheum (2011) 63(3):738–48. 10.1002/art.30183 21305501

[106] Shen H Panchanathan R Rajavelu P Duan X Gould KA Choubey D. Gender-dependent expression of murine Irf5 gene: implications for sex bias in autoimmunity. J Mol Cell Biol (2010) 2(5):284–90. 10.1093/jmcb/mjq023 20802013 PMC2952390

[107] Desnues B Macedo AB Roussel-Queval A Bonnardel J Henri S Demaria O TLR8 on dendritic cells and TLR9 on B cells restrain TLR7-mediated spontaneous autoimmunity in C57BL/6 mice. Proc Natl Acad Sci U S A (2014) 111(4):1497–502. 10.1073/pnas.1314121111 24474776 PMC3910605

[108] Umiker BR Andersson S Fernandez L Korgaokar P Larbi A Pilichowska M Dosage of X-linked Toll-like receptor 8 determines gender differences in the development of systemic lupus erythematosus. Eur J Immunol (2014) 44(5):1503–16. 10.1002/eji.201344282 24500834 PMC4028042

[109] Khan D Dai R Ansar Ahmed S. Sex differences and estrogen regulation of miRNAs in lupus, a prototypical autoimmune disease. Cell Immunol (2015) 294(2):70–9. 10.1016/j.cellimm.2015.01.004 25619140

[110] Dai R McReynolds S Leroith T Heid B Liang Z Ansar Ahmed S. Sex differences in the expression of lupus-associated miRNAs in splenocytes from lupus-prone NZB/WF1 mice. Biol Sex Differ (2013) 4(1):19. 10.1186/2042-6410-4-19 24175965 PMC3843556

[111] Markle JG Frank DN Mortin-Toth S Robertson CE Feazel LM Rolle-Kampczyk U Sex differences in the gut microbiome drive hormone-dependent regulation of autoimmunity. Science (2013) 339(6123):1084–8. 10.1126/science.1233521 23328391

[112] Yurkovetskiy L Burrows M Khan AA Graham L Volchkov P Becker L Gender bias in autoimmunity is influenced by microbiota. Immunity (2013) 39(2):400–12. 10.1016/j.immuni.2013.08.013 23973225 PMC3822899

